# Additive Manufacturing of Earth-Based Materials: A Literature Review on Mortar Composition, Extrusion, and Processing Earth

**DOI:** 10.3390/ma17010202

**Published:** 2023-12-30

**Authors:** Douglas Rocha, Paulina Faria, Sandra S. Lucas

**Affiliations:** 1 CERIS, Department of Civil Engineering, NOVA School of Science and Technology, NOVA University of Lisbon, 2829-516 Caparica, Portugal; 2 Department of Built Environment, Eindhoven University of Technology, 5600 Eindhoven, The Netherlands; s.s.d.o.lucas@tue.nl

**Keywords:** 3D printing, earthen products, clay-based mortar, eco-efficiency

## Abstract

Increasing concerns about global warming and its impact on the environment reinforce the need for new materials and technologies. Additive manufacturing has become more relevant due to its potential to build sustainable and more energy-efficient constructions. However, the materials employed within the technology are not yet fully sustainable. Researchers employing clay as the main binder have found that, besides protecting the environment, it benefits passive control of indoor temperature and relative humidity and contributes to comfort. The mortar design as well as the necessary technological adaptations for the 3D printing of earth mortars are addressed. From a material perspective, this paper reviewed and analyzed the recent developments in additive manufacturing of clay-based mortars, highlighting the main gaps and providing recommendations for future developments in this field.

## 1. Introduction

Earth is one of the most ancient building materials. Many millennia ago, men started mixing earth with water, creating a mortar they used to fill the spaces between small branches employed to make shelter. The space of the former branches and fingerprints of the hands of men who applied them can be observed in archaeological remains [1]. Many buildings built using earth with different technologies have proven their durability and are still in use today. Problems appear when there is a lack of maintenance or, in the worst case, when construction and repair are substandard. This was unfortunately proven in the 2023 Moroccan earthquake, where many earth-based buildings suffered no significant damage, while other buildings, both earth-based and concrete-based, collapsed [2]. It is estimated that 8% to 10% of the world’s population lives in earth-based housing and an average of 20% to 25% in developing countries [3]. It represents a rapid decrease from the previous estimation of one-third of people living in earthen dwellings [4]. Many of these houses are constructed using millennial techniques such as monolithic walls built mainly with cob or rammed earth, or masonry walls using adobe, filling techniques such as wattle and daub, and coating technologies such as plastering and rendering. There are also more recent wall-building techniques, such as compressed earth block masonry, starting in the 20th century [5], and earth concrete, mostly from the 21st century [6].

The diversity of earth technologies and construction systems is linked to the varying material composition of the earth, extracted from the soil, its granulometry (with all fractions or after removal of coarser fractions), its consistency when mixed with different contents of water (liquid, plastic, granular), the way it is compacted, compressed, extruded, or just molded, and cultural characteristics. For pouring earth concrete, a formwork is filled with an almost liquid earth mortar. Alternatively, adobe and cob are techniques suitable for plastic earth mortar consistencies. To produce adobe for masonry, earth mortars fill masonry block molds, the molds are removed after drying shrinkage, and air drying continues until the adobe is ready to be layered using a masonry bedding mortar to produce the masonry wall [7,8,9]. To build a cob monolithic wall, the earth mortar is produced with plant fibers and, while fresh, portions are stacked upon each other to build the wall [8]. Generally, the fibers improve the cob’s tensile strength and reduce shrinkage [8,10], with no need to use formworks. Granular earth is associated with rammed earth and compressed earth blocks. Rammed earth involves using a formwork where layers of humidified earth are compacted, layer by layer, until filling the whole height of the formwork. Traditionally, the local earth was used without removal of coarse fractions and no additions and, for defensive structures, with the addition of air lime [8,11].

Traditional construction techniques employed in the construction of earthen buildings have seen limited development over the years. According to the CRATerre wheel on raw earth applications [4], there are 12 main earth construction techniques, most of them relying heavily on manual work. Nowadays, there is a discussion about the potential to modernize and automate construction methods by prefabricating elements and mechanizing construction processes. Companies and researchers have been striving to bring innovation to traditional earth construction techniques by using easier formworks and equipment that reduce manual work and/or incorporating digital tools, such as the prefabrication of rammed earth walls [12,13,14,15,16], more industrialized earth blocks, earth-based material extrusion, mechanical stacking of clay material [17,18], coating surfaces by spraying earth [19,20], and computer arrangement of ceramic blocks [21]. Some advantages and some drawbacks are associated with different technological approaches.

The industry faces new challenges as the population grows and the environment degrades. The increasing demand for affordable, environmentally friendly, quickly constructible, and efficient houses is pressing the construction sector to innovate. Additive manufacturing (AM) has the potential to speed up the construction process, reduce labor and manufacturing costs, and decrease raw material waste [22]. AM construction can be considered more efficient and ecological than regular construction due to the controlled use of materials. However, to achieve a mechanical performance comparable to conventional formwork construction, a large amount of binder has been used. The limited use of coarse aggregates is the primary reason for this [23].

Material extrusion (ME) is a common type of AM applied to fabricate large-scale elements based on a layer-by-layer construction. This approach makes it possible to construct complex elements in the absence of formwork. In the past two decades, there has been significant progress in the development of material extrusion for structure design, often referred to as 3D printing cementitious mortars (3DPCMs). While cement provides high mechanical performance to mortars and is widely available in many countries, it also has a substantial environmental footprint. It is estimated that new reinforced concrete construction produces between 17.8 and 40.1 kg/m^2^ of waste [24]. The development of new construction techniques should aim to preserve natural resources and minimize environmental impact.

Sustainable alternative materials for ME are under study, and earth (composed of different fractions of clay, silt, and sand after crushing, sieving, and removal of coarser particles) has been demonstrated as a highly eco-efficient solution. The potential to apply this type of local and non-industrialized material may have a big impact on costs and, surely, on sustainability by reducing transportation distances and energy consumption. Furthermore, earth is often available as waste resulting from excavation during building and construction in general. Unfortunately, in many countries, earthmoving materials are often classified and treated as waste products [25], which should only occur when excavating from contaminated areas. However, it is important to recognize that earth possesses a high degree of versatility and can be utilized more effectively and intensively in buildings. Apart from the low energy associated with transport, crushing, and milling, there is no energy used for calcination [6,26]. The labor costs depend on the country’s social and cultural aspects, as well as on building technology. For instance, in regions where earth construction is common and the supply of skilled workers with master technical expertise is high, the cost of labor is lower than in countries where it is seen as a niche market. However, the majority of earth-building technologies are associated with high labor intensity, which can be a barrier to the higher use of earth in building.

Companies like WASP using ME have archived large-scale construction of earthen structures [27,28,29,30], including the creation of a one-story commercial building [31]. Furthermore, successful constructions [32,33] and a house for cohabitation in the time of COVID [34] were printed by the company Emerging Objects. The technology of 3D printing proves to be an excellent method for constructing earth-based houses or producing building elements. This choice of earth-based construction can impact various levels, including the environment, social aspects, and overall efficiency. This emerging technology has been gaining increasing interest and applications over the past few years, with characterization of materials, products, and processes being gradually improved. Most of the projects provide insufficient information on mortar properties (e.g., fresh state and hardened state), the interaction of the mortar with the system (e.g., design path, pump, print head, and robot), and the performance aspects (e.g., mechanical behavior, thermal insulation, ventilation, hygroscopic performance, environment and economic performance).

There are many proofs of concept and prototypes that claim earth extrusion to have a better performance but do not present sufficient data to support the claim [35]. The insufficient details make it impossible to comprehend and compare the results or replicate the tests. Furthermore, local earth is one of the most common materials reported by researchers, but there is a lack of extensive characterization of clay content, plasticity, and mineralogy. It is also non-consensual regarding material plasticity and pumping systems, making it impossible to categorize different approaches related to material stiffness. The lack of information and non-uniform approaches make the development of the technique difficult.

This review article focuses on the application of additive manufacturing of clayey earth mortars used in large-scale construction. It comprises three sections. Firstly, it compiles data obtained from a literature review concerning the materials used, printing systems employed, and laboratory tests reported. Secondly, it analyzes the printability properties of earth-based mortars, encompassing aspects such as pumpability, extrudability, and buildability. Finally, it delves into discussing the eco-efficiency of 3D-printed walls and blocks. This paper aims to promote a better understanding of sustainable construction using clayey earth mortars and to encourage future developments in functional 3D-printed solutions.

## 2. Review Methodology

This literature review was conducted using the online databases of Web of Science, ScienceDirect, Scopus, and Google Scholar. Keywords have been selected and then combined using Boolean operators to obtain a more complete view of the research, for example, “additive manufacturing earth”, “3D printing earth-based mortar”, and “3D printing clay”. The complete list of terms is presented in Table 1. The criteria adopted were similar to those used by Gomaa et al. [35] to find the relevant data, with the conditions described as follows:

- Only publications between 2013 and 2023 (inclusive) were considered relevant; previous studies were excluded due to the existing technology gap.
- Only publications written in English were reviewed.
- The reviewed work must have been focused on material extrusion of clay-based mortars; other digital manufacturing approaches or review papers will only be briefly mentioned.
- The reviewed work must address actual building components/prototypes, either on a small scale (e.g., modular components, bricks/blocks) or on a large scale (e.g., full-size walls); cladding, artistic, furnisher, and non-functional pieces are excluded.
- Only clayey soil/earth are considered; sand, salt block, and so on are excluded.
- Calcinated clay, nanoclay, and geopolymers are excluded from this review.
- Focus has been placed on work that involved actual experiments rather than pure theoretical work; theoretical approaches or review papers will be mentioned, but not discussed or analyzed at significant length.

**Table 1:** Searching terminologies for the literature review.

Terminology	Combined Terminology
Additive manufacturing	Additive manufacturing AND (earth OR clay OR cob OR mud) AND ((based AND material) OR based mortar)
3D printing/3D printed, 3DP	(3D printing/3D printed, 3DP) AND (earth OR clay OR cob OR mud) AND ((based AND material) OR based mortar)
Material extrusion	Material extrusion AND (earth OR clay OR cob OR mud) AND ((based AND material) OR based mortar)

The search, including the criteria, yielded 52 documents associated with the proposed topic. There are a variety of published works, including journal articles, conference papers, and online resources. Table 2 summarizes the selected papers according to the criteria adopted and key data information about each project: explored aspects, source, location, group/company, material, type of clay, fibers, and additions. Table 3 summarizes the selected papers, focusing on theoretical approaches and review papers based on the adopted criteria, providing key data information about each project: explored aspects, source, location, group/company.

When analyzing Table 2, the properties, such as the workability and buildability of mortars, are cited by most of the publications, contrary to the environmental performance, which is hardly analyzed. A lack of interest in the functionality of earth materials is noticed. Only a few studies mention thermal performance, but the results and test procedures are missing in half of the cases. Functionalities such as acoustic, hygrothermal, and ventilation performance have been understudied. It becomes evident that various groups and companies are continuously publishing new developments or continuing studies. Also, new research groups worldwide have begun investigating the printability of earth materials. This consistent stream of research applications and the growing number of research groups reflect the strong interest in sustainable materials in modern construction techniques. At least 30% of the reviewed literature consists of online articles or media publications. These non-scientific publications provide limited information and only a small portion of laboratory research results. It is also possible to verify that there is a lack of information on the earth material, fibers, and additions. Earth materials were adopted for 75% of the experimental research, which supports the sustainability of using locally sourced raw materials. The main issue with the literature is the lack of thorough material characterization. The formulation of mortar is significantly influenced by the type and proportion of clay in the earth being used. Illite and kaolin clays are commonly selected for mortar production due to their relatively low shrinkage when compared to montmorillonitic clays [77]. The characterization of clay content and type is notably lacking in nearly 60% of the cited references. Additionally, there is limited information provided in the literature regarding parameters such as size distribution, plasticity index, liquid limit, and plastic limit. This lack of a comprehensive characterization can hinder a thorough understanding of the materials used in the studies.

Shrinkage can lead to the development of cracks, which can negatively impact both the mechanical strength and the aesthetic quality of plastering mortars. To mitigate dimensional variations, additional aggregates can be introduced to the earth’s composition [6]. Moreover, various additives and admixtures can be incorporated to enhance the properties of earth mortars. Natural fibers and oils, such as straw and linseed oil, as well as binders like hydrated lime and gypsum, are frequently employed as common stabilizers [78,79]. While natural hydraulic lime and cement are also utilized, they may not be deemed necessary or as compatible as the previously mentioned options. This is mainly because, in the quest to produce low-embodied energy mortars, other natural solutions like biopolymers are gaining popularity as stabilizers [47,80,81].

Analyzing Table 3, the bibliometric collection has general information about different techniques, including additive manufacturing with clay-based mortars. The review papers offer limited information about the material properties. Theoretical approaches or proofs of concept represent 50% of the selected documents. The limited number of studies addressing the thermal performance of printed solutions is noticeable. Due to the high thermal performance of earth-based constructions, further developments on this topic should be addressed. The theoretical approach conducted by the A.C.T. group [67] is the only study that focuses on environmental assessment. Other authors mentioned the potential but missed the quantitative impact of printing with earth.

## 3. Processing Earth Material for Additive Manufacturing

### 3.1. Earth-Based Mortars Used in Additive Manufacturing in the Literature

#### 3.1.1. Fresh and Hardened Properties

The Institute for Advanced Architecture of Catalonia (IAAC) was one of the first to use additive manufacturing of earth materials through the initial projects Pylos, TerraPerforma, and Digital Adobe. Pylos mortar contained 96% earth and an addition of 4% natural additives in order to improve its strength and viscosity [39]. The material is described as relatively weak with high shrinkage performance (>6% of the initial extruded size). The next project of the same group was TerraPerforma, where the material was described as “mud stabilized with fibers, sand and proteins”. It was a mortar with non-uniform shrinkage but higher than 7% of the initial extruded size and low strength (compressive strength < 0.2 MPa after 6 h) [41]. Digital Adobe was the tallest project executed by the IAAC group; it involved assembling modular blocks made of mortar based on clay, water, aggregates, and bio-based additives that were previously printed and cured. The 20 mm × 20 mm × 100 mm block specimens achieved a flexural strength of 5.01 MPa for a three-point bending test [82]. The projects performed by the IAAC group demonstrate how the combination of clay and other materials to enable extrusion allows for the creation of optimized infill objects that could improve thermal and acoustic performance. The lack of information concerning material ratios and mineralogy makes it difficult to comprehend the results related to mechanical performance and shrinkage. The absence of composition information makes it impossible to replicate the same mortar and verify the test results. Equally important are the details about test conditions and the drying process, which should be described to ensure reproducibility and the development of the technique. Many research groups prefer to showcase the concept and final products while omitting crucial information about the system and materials used. Consequently, this hinders the complete mastery of the process of printing earth-based mortars. Complementary laboratory test data gathered from the review of the literature are presented in Table 4, which offers a concise overview of each project, presenting key data information organized by year of publication; mineralogy or chemistry analysis; rheological investigations; fresh properties; hardened properties; and pump system.

Table 4 shows that XRD or XRF analysis of clayey earth is only mentioned in less than 15% of the literature, and the same percentage of studies look at both fresh and hardened properties. As previously mentioned, the characterization of the materials is indispensable for comprehending the results.

The number of research publications related to ME with clay has been increasing in the last 10 years. However, there is still a lack of standardized tests and parameters to evaluate the printability of earth-based mortars. The few existing standards and control parameters for traditional earth construction methods are challenging to apply to additive manufacturing [63].

Before and during the printing process, workability and rheology are crucial characteristics that control the material flow into the system and its behavior right after the extrusion. Key factors such as dynamic yield stress and viscosity clarify the minimum pressure required to pump the material, and static yield stress provides information about the maximum load that the first printed layer can support. Fresh properties enable quantitative characterization of the material, with nearly 6% of studies presenting a comprehensive laboratory rheological investigation. To produce a printable material, authors frequently use a trial-and-error strategy. In some cases, highly stiff materials are created, which may pose challenges for testing using standard rheometers.

Analyzing the fresh properties, printability is mentioned in all selected references. The printability test involves attempting to print the material with or without mechanical tools. It is distinguished from extrudability and buildability tests because, for extrusion tests, both qualitative and quantitative characterizations are performed. Buildability tests consider factors like geometry and the maximum height a structure can reach without collapsing. The Vicat needle test was reported in the bibliography to define the setting time when additives were added to the mortar. Tests such as the penetrometer, material flow, and slump provide information about the workability of the material; in some cases, it is possible to estimate the yield stress and viscosity. The simple workability tests could be interesting for the standard characterization of the mortar’s properties.

For hardened properties, it is common to perform mechanical characterization with uniaxial compression and flexural strength tests. A problem throughout the publications is the absence of standard procedures to produce printed samples. Due to the limited capacity to construct layers, some authors created samples over multiple-day sessions [64], which can result in weak points between the layers [83,84]. Other authors preferred to build a solid multilayer block [26,44,59], aiming to avoid the collapse of the structure and subsequently cutting smaller samples. In this case, higher weight pressure or voids between layers can be created.

#### 3.1.2. The Impact of Using Stabilizers Other Than Fibers

Clayey earth is a material with a slow drying process, and the mechanical build-up of strength starts to develop after moisture is released. For this reason, additive manufacturing with earth is a difficult challenge. During layer-wise construction, the previously printed layer needs to develop enough strength to support the additional load of subsequent layers. In the literature, various methods of stabilization to enhance the early strength development of earth-based mortars can be found [26,62,85]. However, as previously mentioned, to create a sustainable mortar, the materials should obey the principles of sustainability; so, low quantities of calcinated materials and chemicals must be used. The use of biopolymers, such as alginate (a natural fast-setting biopolymer), has been proven to be efficient in improving the fresh strength of earth mortars and enhancing the buildability of printable mortars [44]. One study [55], employing 3% of the earth mass in alginate and using a rich clay-based earth (>60%), successfully demonstrated the rapid development of yield stress when the polymer was added to the mortar. The mortar’s consistency was controlled by yield stress. However, to safely pump the mortar, the water content was increased. On the other hand, the investigation overlooked shape retention during the curing process. If the plastic mortar contains a large percentage of clay (>25% wt), dimensional variations are highly likely to occur. Large-scale samples are typically more susceptible to shrinkage and crack propagation. Therefore, future developments should focus on producing representative or full-scale samples.

The use of air lime to mineralize vegetal fibers has been employed in traditional cob mortars. A combination of rice husk and lime forms a natural bio-composite that improves the mechanical resistance of earthen mortars [57]. The WASP group has effectively embraced the utilization of hydrated lime, integrating it with rice husk to create a bio-composite rich in silica [57]. Another study [59] exploring printable mortar has demonstrated that incorporating higher amounts of air lime results in a more workable and moisture-retaining mortar. Acting as a binder, the addition of air lime had a positive impact on compressive strength, which was almost proportional to the amount of air lime added [59], although it should depend on the content. Air lime’s significance transcends its material properties, encompassing a sustainable life cycle when compared to cement. It notably undergoes calcination at lower temperatures (circa 900 °C), aligning with environmentally friendly and vernacular conscious practices. Furthermore, it can be recycled or dispersed onto the ground without causing soil contamination. However, when adding air lime to an earth mortar, reusability is no longer possible, as an artificial stone is produced.

Rapid development of mechanical properties is of utmost importance to ensuring efficient constructability and facilitating expedited construction processes. Incorporating limes with hydraulic properties or cement into the formulation notably enhances mechanical performance both in the short and long term, while concurrently improving water resistance [81,86]. However, the primary consideration surrounding the application of these hydraulic stabilizers relates to sustainability. These materials have a high embodied energy and their use as stabilizers results in a larger ecological footprint. Reutilizing such mortars is no longer possible, and challenges in recycling them become evident, subsequently generating waste at the end of life. The stabilization of earth-based mortar remains a noteworthy concern, particularly in the context of the material extrusion process. Earth mortar, on its own, has demonstrated that it is capable of supporting the construction of single-story structures [44,57]. More studies about the fresh and hardened mechanical performance with and without additives can help to understand the type of material that is ideal for common applications. Also, geometry has an important role in mechanical performance. The infill density and the thickness of the wall can significantly affect the structural, thermal, and acoustic performance.

#### 3.1.3. The Impact of Employing Dispersants on Clay

To guarantee the mortar’s extrusion, a higher water ratio is used to maintain its fluidity. However, this increased water content negatively affects the mortar’s properties, leading to higher shrinkage and reducing the buildability of the printable mortar [43,51]. To enhance workability and reduce water content, a compatible dispersant with clay can be employed. Sodium hexametaphosphate, sodium polyacrylate, carboxymethylated xylan, and sodium silicate with various silica moduli have proven to be the most effective dispersants for earth mortars [87]. The use of natural ones, such as tannins, has demonstrated positive results in enhancing flowability as well as improving water resistance [88,89] and mechanical performance [90,91]. The influence of dispersants on the printability of clay-based mortars needs to be investigated, since the reduction in water and increasing flowability align with the goals of extrusion.

#### 3.1.4. The Effect of Incorporating Fibers

Comparing the additive manufacturing process with the traditional manual cob technique, similarities can be observed in their construction processes. The process of building cob walls typically involves layering mortar into thick, solid walls, level by level, which is similar to additive manufacturing. Perhaps this is the reason why there are so many attempts to manufacture cob mentioned in the literature. However, one can discuss if it is correct to refer to walls built using this manufactured process as cob. Cob mortar is a mixture of clay, sand, water, natural fibers, and sometimes additives. The primary differences between manufactured cob and artisanal cob are the thickness of the walls, as they are solid for the latter, the size of the fibers (higher also for the latter), and the consistency of the mortar. For the printing process, the mortar needs to have fluid–plastic behavior [43].

In the publications, a maximum addition of 2% fiber is recommended for manufactured cob [51]. This ratio is essential to prevent pump blockages and clogging of the nozzle [43,59]. The use of fibers has been shown to improve mechanical resistance and reduce shrinkage [57,59], but contradictory results have been published [60,61]. Only a small number of authors exclusively tested the influence of fiber additions; depending on the fiber ratio and other additives, a reduction in compressive and tensile strength was described [61,92]. The combination of a natural stabilizer (potato starch) and sisal fibers negatively influences buildability, causing early collapse of the structure [61]. Since the previously mentioned study focused on compositions with potato starch gel, it is difficult to draw exclusive conclusions about the influence of added fibers.

### 3.2. Pumpability

Pumpability [93] characterizes how effectively the fresh mortar can be conveyed from the pump to the extrusion nozzle section [84]. The rheology of the material significantly impacts force transmission; lower plastic viscosity tends to reduce pumping pressure [94]. Depending on the type of conveying system, it is possible to target the workability of the material. Alternatively, it is possible to adapt the system to the material properties, making this second option more laborious and expensive. There is no universal system, as each research group and company has developed and combined different conveying systems, deposition equipment, and geometric design software. Changing the feeding system without impacting the final result is unlikely because material properties are closely connected. As demonstrated in the research conducted at University Park [59], focusing on manufactured cob extrusion, significant variations in mortar consistency were observed between a manual handheld piston pump and an electric handheld piston pump. Moreover, in adapting to the use of a cavity pump, adjustments in both the water ratio and the formulation were necessary.

**Figure 1:**
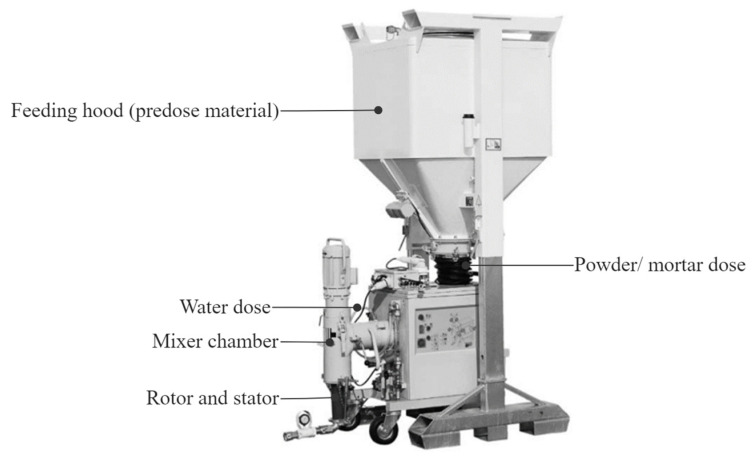
Conveying system integrated with mixing and feeding tank (adapted from [99]).

Several components constitute the feeding system: the pumping equipment, tank, elevation, and transport of materials, mortar dosing, water dosing, mixer, and hose. Several of these components can be incorporated into one machine, making it possible to mix and pump the mortar. This type of equipment reduces human interaction and allows continuous printing. Figure 1 is an example of a mixing pump with an incorporated powder feeder. There are several printers on the market that are specially designed for three-dimensional printing of clay-based materials [95,96,97,98]. Based on Table 4, it is possible to identify the pump systems that are recurrently used: piston pumps (electromechanical, hydraulic, pneumatic) and screw pumps (progressive cavity and Archimedes’ screw). Both systems have their benefits and problems, and it is possible to find solutions on the market that integrate the piston pump to deliver the mortar to the print head and a screw pump at the print head is used to control the extrusion. A printing system composed of two pumps can be used for mortars or plaster, with or without sand granulometry.

The ME process is associated with a fluid–plastic earth; during printing, the material suffers a deformation introduced by the system, and the final shape is created at the nozzle cross-section. To maintain the shape, the mortar needs to have enough plastic strength. The plasticity of the mortar can be controlled by the ratio of clay, aggregate, water, and additives, but also by the type of clay and mixing method [61], but the workability of the mortar needs to be compatible with the conveying system.

Due to the plastic consistency of earth-based mortars, some researchers opted for the use of pumping systems with a piston. In this kind of system, the mortar can be stiffer, with high yield stress and viscosity, allowing it to maintain its shape right after extrusion. It is considered an extrudable mortar when it flows without blocking the system and achieves the right consistency after being extruded. In pneumatic systems, dense mortars can affect the extrusion rate, quality, and consistency of printed building elements [55]. A stepper electric motor is typically used to control the extrusion rate and lower torque, improving the quality of the extruded mortar. This motor can be applied to both pneumatic and electromechanical pumps. A major disadvantage of piston pumps is their limited capacity for continuous printing, as the mortar quantity is restricted by the chamber’s size. The frequent pauses for mortar refilling are time-consuming, particularly when applied to large-scale constructions [43]. Another issue with piston extrusion is mortar segregation; stiff mortars require high pressurization, and this pressure increases their compaction, which can lead to water migration [43,56]. Numerous compositions have to be tested to achieve the balance between the viscosity of the mortar and the required mechanical and structural properties [43]. The use of a progressive cavity pump can be adopted to reduce the shear forces introduced by the extrusion system, preventing segregation and enabling a continuous mortar flow [61].

The duo ram extruder has been proposed [55] to minimize manual interference and enable continuous printing processes. The use of multiple cartridges can enhance productivity and reduce the time required to reload the system, although it necessitates managing a larger number of robots to maintain system automation. Another disadvantage of a rechargeable solution is the potential introduction of air into the cartridge [43]. It is important to note that the use of premixed mortars ensures material quality and conformity, offering greater assurance of the quality of the extruded mortar. Looking ahead, given the limitations of existing commercial piston pump solutions, it is unlikely that large-scale projects can be delivered. The substantial number of interactions, whether human or involving support robots, and the time needed to reload the system can be counterproductive, deviating from the primary goal of digitizing earth construction.

Optimizing mortar rheology for compatibility with the system has been shown to be an effective methodology [44,100]. By setting a fixed pump pressure and predicting or testing the loss of energy introduced by the system, a range of yield stress values that ensure the extrusion of the mortar can be estimated. Using this approach, it is possible to optimize mortar properties for use with a specific pump. While controlling dynamic properties can affect the quality of the extruded element, the addition of more water or plasticizer can decrease its buildability [43,61]. Pumpability is a crucial part of printability, but many other aspects must be addressed, including hardened properties. On the other hand, control of the maximum yield stress is not always linear. When different additives are incorporated, mortars with higher yield stress can be extruded with the same pump. The addition of fibers can lead to an increase in the shear rate. The decrease in workability caused by the addition of fibers is compensated for by increasing the water ratio in the mortar [61].

Screw pump extruders have been proven to be effective solutions for supplying mortars with different compositions, whether they include fibers or not [44,56,57]. When using a mixing pump, there are two types of mixing procedures: dry materials or pre-mixed mortar. The primary advantage of screw extrusion lies in its ability to provide a continuous flow of material. Therefore, the quantity of the material is not limited by the size of the equipment; it is possible to add or produce more mortar during the printing session. However, all systems have negative aspects. The main problem with this system is the maximum size of the aggregate or the proportion of the fiber. To avoid clogging the system, proportions need to be controlled.

Many other factors can be added to the characterization of the material pumpability, including the lubrification layer [94,101] and segregation [26,101], which influence the quality of the extruded material. Additionally, the fabric material, diameter, and length of the hose impact the pump pressure due to friction forces and mortar viscosity. Calibration between the print head movement and the extrusion speed has a significant impact on the outcome [40]. Some authors have suggested studying the pumpability using common tests like flow table, slump, penetration, and slug test (measurement of mortar droplets at the nozzle), along with more advanced testing employing robust devices such as viscosimeters, rheometers, sliding pipe rheometers, and ram extruders.

### 3.3. Extrudability

The nozzle is the final component of ME, shaping the mortar as it is pushed through this section. A standard cross-section is normally used at the end of the printhead, but other gadgets can be incorporated into the system to improve the material performance. These components can pulverize liquids, introduce additives, or reinforce the mortar. Just before the nozzle, a mixing chamber with a screw could be used to manage low-viscosity and low-yield stress materials, combining the mortar with an activator to enhance shape retention. Alternatively, when dealing with stiff mortar, local vibrations can be applied to improve workability and reduce extrusion pressure. There is also the option of introducing moisture to the extruded material during or after printing. This can be achieved through the dispersion of water mist or by utilizing chemicals to accelerate mortar setting [101].

**Figure 2:**
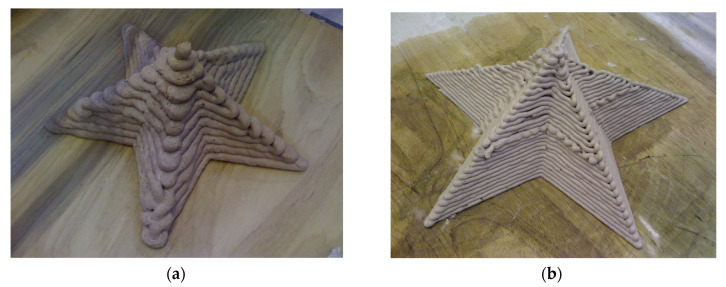
Comparison of resolution for printed gypsum mortar using different nozzle [104]: (**a**) 22 mm by 15 mm; (**b**) 9 mm by 6 mm.

The section must be compatible with the mortar’s composition and consistency. If the section is too small, there is a risk of clogging and segregation [23,101]. To mitigate this risk, the maximum aggregate dimension and fiber length need to be controlled. As proposed by Buswell et al. [84] and Mechtcherine et al. [101,102], the nozzle shape can be divided into two types according to its section: elliptical/round or rectangular/square. The contraction section can incorporate trowels capable of shaping the material and achieving smooth surfaces, most well-known as “contour craft” [103]. The resolution of the printed object is directly linked with the size of the nozzle; small dimensions achieve better freedom of geometry and resolution [104,105], but they are directly linked with the building time and complexity of the object. The impact of using two different nozzle sizes on the printing quality can be observed in Figure 2, where a larger nozzle yields a rougher surface.

**Figure 3:**
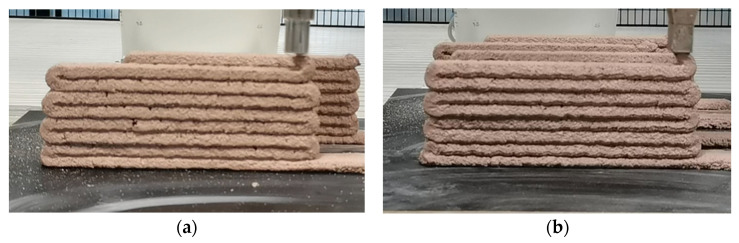
The influence of the nozzle section on the micro-cracking during the printing of clay-gypsum-pozzolan mortar: (**a**) round cross-section; (**b**) square cross-section.

In Figure 3, the printing quality is associated with the nozzle type. When using a nozzle with an elliptical section, the extruded layer may deform at the boundary due to the lack of support material, causing micro-cracking [106]. To prevent deformation after the extrusion, the material strength needs to be higher than the gravitational flow [101]. For printable cement-based mortars, it is possible to define a minimum yield stress that satisfies the condition of 3DPCM. A similar approach needs to be developed for earth-based mortars. Currently, there is a gap in testing protocols and rheological properties of these materials [63]. Nozzles with a rectangular section can avoid cold joins and provide better adherence for the subsequent layers due to a larger contact area with the previous and following layers.

**Figure 4:**
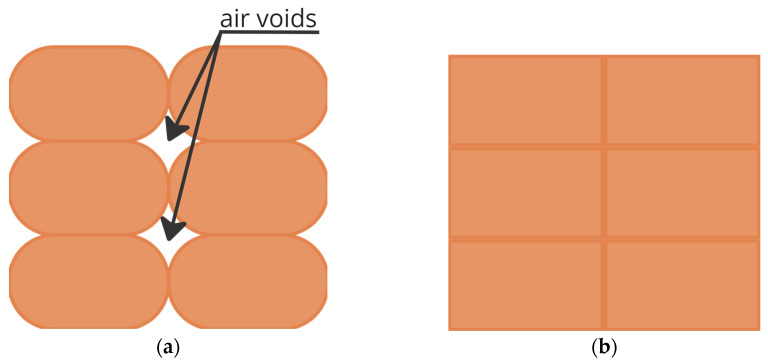
Representation of air voids for different nozzles—cut section of printed samples with two layers side by side and four layers in height of earth with alginate mortar: (**a**) printed with a circular cross-section; (**b**) printed with rectangular cross-section.

Perrot et al. [44] observed a higher percentage of air pockets using a circular nozzle, as represented in Figure 4, when using a stiff clay-based mortar (shear yield stress of 1.5 kPa). The extrusion of stiff material can be likened to the “infinite brick regime” [93,101], where the filament has almost zero deformation. Due to the low flow of stiff mortars, higher pressure is required during the pumping process.

**Figure 5:**
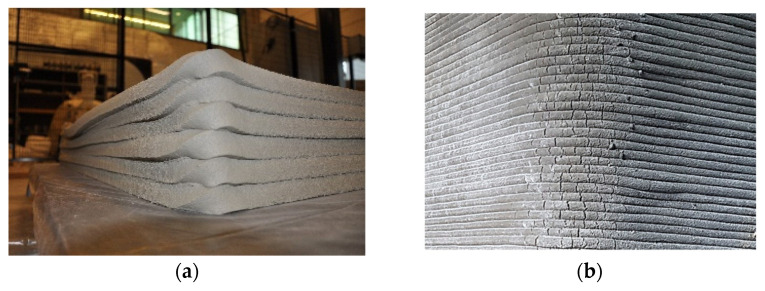
Printing quality problems with rectangle/square nozzle section: (**a**) twisted filament [108]; (**b**) tearing and cracking of printed curve layers [109].

The deposition system can influence the type of nozzle. Three-axis robots have more constraints in movement; in most cases, the print head is restricted in one direction. For systems with limited movement, a round nozzle is preferable, as it allows changes in direction without necessitating the rotation of the nozzle. The printing path for robots must consider both the geometry and the selected nozzle. The necessary radius for creating curved objects can also dictate the nozzle geometry. A rectangular section increases the radius of curvature compared to a round nozzle. Small cracks in the outer edge caused by curvature radius reflect on the quality of the printing, strength, and buildability [107]. Figure 5 illustrates two common problems that frequently happen with rectangular nozzles when changing direction. The geometry of the object can influence shrinkage, as observed in the TerraPerforma project [41]; depending on the pattern and radius, resistance to cracks can be improved. Analyzing the bibliography, a gap was identified in the analysis of large-scale printed objects produced with clay materials in terms of their drying and shrinkage effects and mechanical performance. Smaller samples produced in one direction are less representative of real constructive elements. The exposed area during drying and the lack of constraints, due to the absence of formwork, leads to high dimension variation and consequently cracking propagation.

### 3.4. Buildability

The building rate and the stability of the set of layers are dependent on the maximum load the first layer bears, the early strength development, and the printed element geometry. Assuming a fixed toolpath and nozzle size, the material flow and print head movement speed are the main factors in determining the pace of construction during a printing session [110]. Maintaining an optimal production rate relies on a uniform mortar feed and compatible layer deposition. The time required for this process varies based on the project’s geometry, potentially ranging from a matter of seconds to several minutes for the deposition of each layer. One of the main benefits of using this manufacturing process is the speed of construction and low human interaction. However, the use of mortars with low fresh strength can affect the time necessary for construction. Materials like clay-rich earth, which do not undergo a chemical reaction for hardening, depend on the drying process with the exchange of moisture with the surroundings; during this process, the mechanical resistance of the mortar is relatively low. Experiments involving the continuous layering of printed mortars can serve as a valuable approach to estimating the maximum number of layers and the time required for the conclusion of an entire building or wall segment.

Analyzing Table 4 reveals that the buildability test is the most commonly employed approach among academic research groups. Nevertheless, in the study of layer stability, different protocols are adopted by researchers due to the absence of standardized procedures. Notably, the printing rate, mortar flow, layer dimensions, initial timing test, and various other characteristics show variations across the array of published works. Depending on the consistency and composition of the mortar, for the same system, it is possible to obtain different printing speeds. For instance, Veliz Reyes et al. [43] reported a printing speed of 5 mm/s for cob mortar and 15 mm/s for earth mortar, differing by the fact that the first included natural fibers. The calibration of the print speed and the mortar flow is normally described as the extrudability; it can be tested by a visual and quantitative method combined. This test aims to verify the continuous extrusion of filaments with a satisfying quality and non-dimensional variation in the layers [100].

After determining the printing speed, the next phase of the testing procedure involves evaluating the buildability of the mortars. This step entails conducting various tests to predict the maximum number of layers that could potentially lead to structural collapse. Researchers [100,111,112,113,114] often mention two distinct geometries for these tests: the cylinder and the single-direction wall. However, it is important to note that the specific test protocols vary in the literature, making it possible to cautiously compare the number of layers, layer height, and production time for different mortars and compositions. In the case of simple stability settlement tests, the geometry’s failure is of lesser significance due to the minimal or negligible alteration in the extrusion path. Often, what is observed is the plastic collapse of the structure, specifically the point at which the first layer reaches its maximum load resistance. The initial resistance of the layers is linked to the static yield stress of the material. A higher yield stress or rapid flocculation proves advantageous for a material intended for 3D printing [115]. Controlled protocol tests (non-standard with calibrated equipment and easy to replicate) could be adopted to evaluate the building capacity of the fresh mortars, such as the unconfined compression test [44,116,117,118,119] and ultrasonic pulse velocity test [117].

A maximum wall height of 0.58 m was successfully achieved with a cob mortar (earth with vegetal fibers) [55]. Similarly, in the Digital Adobe project [46], a comparable height of 0.60 m per day was attained, although a more conservative limit was adopted during the printing sessions. In the case of earth mortar without fibers [44], predictions indicated a maximum layer height of less than 1 m per day. However, when fast-setting alginate was introduced, the time required to develop the strength necessary to support a 1 m high wall was remarkably reduced to approximately 6 min. The time consumption for building with earth remains a significant concern for the use of some technologies, and the introduction of different additives to accelerate the development of fresh mechanical resistance needs to be applied in future studies. Additionally, the environment in which this technology will be applied must be taken into consideration, given different weather exposures and curing processes.

Project Gaia [28] serves as an example of a single-story circular building featuring a wooden frame ceiling. This project offers a significant demonstration of the unique challenge posed by 3D printing technology in a Mediterranean climate using a mortar composed of clayed earth taken from the site, straw-chopped rice, rice husk, and hydraulic lime. The dimensions of this structure span 320 square meters in the plan. Despite the inherent complexities of 3D printing in an outdoor environment, the construction of this sizable building was successfully accomplished within 10 days. Casa Covida [34] is located in the high alpine desert of Colorado. Due to the climate conditions, it is possible to build 30 cm per day; this rate of production requires careful resource allocation and optimization [74]. The project utilized the fourth axis rail system, creating a robust structure onto which the 3D printer was moved after each printing session, typically covering a height of around 400 mm. This approach allowed for the mortar to be precisely deposited and then subsequently left to dry and harden under the natural elements of sun and wind [34].

Another important consideration when slowing down the building rate is the adhesion between layers. Recent studies on cementitious materials indicate that adhesion between two layers decreases over time [65,71,120]. It is expected that similar issues may arise with earth-based mortar, as adhesion with the previous layer is likely to reduce during the drying process. However, the effect is expected to be less significant in comparison to cement mortars. Further investigation is needed to confirm this hypothesis.

Aiming to minimize costs and material waste, unconventional wall configurations involving external and infill layers are employed. This pursuit leads to the development of an optimal toolpath, effectively enhancing the buckling stability of the structure [53]. Until now, only blocks and small samples have been tested. There is a need for more research projects addressing optimal printing for earth mortars and building elements.

## 4. Eco-Efficiency of Printable Earth-Based Solutions

### 4.1. Eco-Efficiency of Earth Building Products

Earth, being soil after excavation, is one of the most abundant raw materials in the world. It can be reused indefinitely for different purposes simply by adding water and regaining its workability. To accomplish this, the addition of chemicals, such as common binders, must be controlled or compatible with product reuse [6] and recycling. As mentioned before, earth is a natural material that can create a sustainable cycle that consumes little energy and generates almost no pollutant emissions. The use of earth building products and elements benefits comfort, aesthetics, and ecology, reducing energy consumption. Earth-based products can offer, by their nature, a positive influence on fire protection, acoustic and hygrothermal performance, and thermal inertia [121]. In comparison with cementitious plaster systems, earth plasters have a superior capacity to cyclically store and release water vapor [122]. Consequently, earth building products can passively contribute to reducing the fluctuation of relative humidity in indoor spaces [6,123]. Another issue being addressed nowadays is the increased capacity of earth products to capture pollutants, namely CO_2_ [124] and ozone [125], in comparison to other products.

The environmental advantages of clayey earth mortars in comparison to common mortars have been quantified by Life Cycle Analysis (LCA) [126]. Focusing on CO_2_ emissions, earthen mortars have relatively low energy consumption and low air pollutant emissions [126].

### 4.2. Sustainability of Additive Manufacturing Earth

With the fourth industrial revolution, the construction industry started to invest in automation, using robots and mechanical arms to build faster, safer, and cheaper [24]. In the beginning, the principal material employed by this emerging industry was cement, due to its good mechanical resistance, achieved in a short period of time. As mentioned previously, concerns about the use of cement as the main binder for additive manufacturing are now under debate, since the quantities of clinker are higher than those of traditional in situ reinforced concrete. The industry has proposed alternative solutions that would allow for the partial replacement of cementitious products with other materials and residues. Supplementary cementitious materials (SCMs) such as silica fume, fly ash, and slag that can be used as a partial replacement of cement for 3DPCM have been studied by some authors [94,100,111,116,127,128,129]. Even though the partial replacement of clinker by industrial wastes can improve the ecological impact of the mortars, 3D mortars are still heavily dependent on cement due to the small quantity of replacement for SCMs [23]. There is a limited supply of SCMs throughout the world, which makes it difficult to produce more sustainable cement mortars on a large scale using these materials [130]. With the modernization and introduction of new techniques, the new materials must be environmentally friendly but also designed for high performance and quality. To achieve these principles, cement should be reduced to a minimum, and use of environmentally friendly materials must be prioritized. Additive manufacturing with earth-based materials seems to be a viable, economical, and environmentally friendly solution.

The attempt to digitalize earth is relatively new, but it is noticeable that there are a growing number of studies and investments in this field [35]. The exponential work confirms the potential and the future industrial perspectives for the use of earth as a material. From a sustainable point of view, it is possible to compare 3DPCM with conventional cast concrete solutions [24,67,131,132,133,134], and even more, it is possible to compare 3DPCM with 3D Printing Earth-based Mortars (3DPEM) [67]. However, LCAs are difficult to compare due to the different parameters and considerations included by the different authors. For this reason, there is still not a consensus that 3DPCM is more sustainable than conventional mold concrete solutions. Comparing the CO_2_ emissions, 3DPCM is 91% worse than three-dimensional printed cob (3DPCob) [67]. Global warming value consideration came from clicker production, which requires a high temperature (around 1500 °C) [112], consequently releasing tons of gases into the atmosphere. Instead, cob mortar, a natural raw material with no heat treatment and normally locally available, reduces the production of greenhouse gas emissions to almost zero if any mechanical equipment is used. However, that is not the case for additive manufacturing.

Processing earth could follow similar traditional earth building processes where intensive labor is replaced by robots or modular manufacturing products. Following the same principles as 3DPCM, the studies with 3DPEM and 3DPCob have been exploring the possibility of using clay as the principal binder for additive manufacturing. Processing clay by the extrusion of filaments is one of the most used processes, due to the possibility of using a fresh material without the necessity of drying or formwork. At the market, it is possible to find small-scale three-dimensional printers dedicated to clay material or large-scale projects that use earth-source mortars.

Using materials and products with low embodied energy is a viable solution to reduce the ecological impact of buildings in the future. The eco-efficiency of additive manufacturing associated with sustainable materials and products has a significant positive impact on the environment. In comparison to 3DPCM, 3DPEM, and 3DPCob, producing less material waste is feasible due to the possibility of reusing the mortar. However, this is only achievable if the additions are compatible with recycling. Another advantage of using earth mortars is the extended open time, which reduces the blockading or clogging of dry material inside the hose. However, drawbacks of printing earth mortar include lower mechanical resistance, buildability, and water durability compared to cementitious printable mortars.

### 4.3. Functionality of Printable Earth Building Walls

Indoor conditions, such as the indoor air quality (IQA), relative humidity, and temperature, play an important role in determining the quality of life and building preservation. There are several advantages to living and working in a healthy environment, including a higher level of productivity, improved sleep, and reduced stress [135,136].

TerraPerforma, an IAAC project, used Energy2D and a customized two-compartment test with a heat source, thermal sensor, and thermal camera to study the thermal performance of single-printed unfired blocks. The test demonstrates the efficiency of the hollow geometric design to reduce heat transfer. For this assumption, it was compared to two different walls made with the same amount of material: a standard wall, partially filled with the same material; an optimal wall solution with hollow geometry; and a customized surface. To design the optimal wall solution, a Ladybug tool for solar radiation simulation was applied; the final design allowed maximizing 436% of the radiation during the winter and minimizing the radiation gains by 70% during the summer [41]. The use of the thermal inertia response of earth-based products in combination with geometry can benefit indoor conditions. This study demonstrates that, with the same material, it is possible to optimize the storage and dissipation of heat. With an optimal solution, it is possible to use small quantities of raw materials and produce eco-efficient products that contribute passively to temperature control.

In terms of thermal conductivity, the addition of fibers can be beneficial, as these lightweight materials can reduce the dry density of mortars [60,92], which decreases the material’s thermal conductivity. Moreover, a higher content of fibers has been shown to affect the stability of the structure during the printing process [60]. A comparison was made between the thermal performance of 3DPCob wall samples and manual cob wall samples [51]. For the 3D-printed samples, subsoil from farmland in Cardiff, UK, was used. Four different printed solutions were produced: solid wall; double-layer wall with a continuous air gap; triple-layered wall with air pockets; and double-layered wall with pockets filled with straw. These printed samples were compared to the manually fabricated cob samples using a Heat Flow Meter. As expected, when comparing the 3D-printed solutions, the solid sample shows the lowest conductivity and higher density, followed by the double-gap and single-gap solutions, respectively. The best conductivity results were obtained for the double layer filled with straw. It is important to highlight that the thickness of the manual samples was less than 22% of the 3D-printed samples. However, the sample with the lowest conductivity results was found to be a molded one. As the author suggests, a more accurate comparison between the two techniques could be made if the samples were created using the same material and with the same dosages. This study confirms that additive manufacturing can be used to produce solutions with low thermal conductivity, which can benefit earth-based houses.

The ventilation of printed earth-based walls opens avenues for exploration through the strategic creation of channels and geometric arrangements. These configurations aim to enhance the circulation and pressure of air within the material, potentially offering a means to passively manage indoor air quality. By cleverly combining thermally conductive materials with well-designed airflow channels, the temperature of the enclosed space can be influenced, contributing to overall thermal comfort.

The optimization of inner geometry, openings, and microperforations can be efficiently achieved through the application of computer fluid dynamics simulations. Tools such as RhinoCFD have proven effective in simulating and designing printed walls, allowing for refined assessments of airflow dynamics and their impact on the indoor environment. Through these simulations, the potential to fine-tune channel arrangements and design specifics emerges, further advancing the integration of passive ventilation strategies in printed construction [41]. The geometry of the modular segments can be used to design cavities that allow the air to circulate and consequently influence the ventilation conditions [137]. The inventive prowess of the POTplus group is exemplified through their project “Common action walls” [138]. Within this initiative, they conceptualized modular rammed earth blocks that, when meticulously assembled, dynamically evolve into a permeable wall. This ingenious design showcases not only the artistry of structural transformation but also the functional potential of adaptable architectural elements. In a parallel vein of innovation, Emerging Objects created the prototype “Cool Brick” [139]. This project delves into the intricacies of material composition and geometric configuration to craft a brick that allows air to circulate, cooling indoor spaces. Simultaneously, these bricks serve as reservoirs for water vapor, strategically releasing stored moisture to facilitate the cooling of indoor environments. The use of the moisture buffer capacity of the materials is one passive mechanism that can regulate the relative humidity (RH) of the indoor air and consequently control the temperature [140,141,142]. As mentioned before, clay possesses a remarkable ability to absorb and retain moisture, subsequently releasing it gradually as internal pore pressure increases [143]. The difference is significant when earth mortars are compared to other mortars [144].

The creation of voids and channels is a difficult task due to the self-sustention of successive layers when the material is in the fresh state. This can influence the productivity and stability of the structure. Within existing literature, it is possible to find a solution involving the utilization of temporary support materials that can be removed after curing [110] or the use of bioproducts that can be incinerated, leaving voids where the bioproduct was placed before its combustion [145].

Utilizing the innate porous structure of materials offers an opportunity to capitalize on sound insulation. It is widely recognized that materials possess the capability to absorb sound waves, depending on their reverberation capacities. When sound interacts with materials, energy is dissipated. Notably, panels housing cavities exhibit enhanced sound absorption abilities compared to their uniform equivalents from the same material. An example in this context is the “Involute Wall” design by Emerging Objects [146]. In this project, wall blocks are manufactured by using resin and sand, and are characterized by their substantial mass and cavities. By intricately designing these blocks with cavities, the structure gains heightened sound absorption potential, serving to mitigate the propagation of unwanted noise [146].

The inclusion of self-sensing properties has been explored by incorporating various fillers such as carbon fibers [147,148,149], carbon microfibers [150,151], carbon nanofibers [152,153], carbon nanotubes [153,154], and graphene nanoplatelets [155]. Utilizing carbon fillers allows for a piezoresistive behavior capable of identifying critical conditions like strain concentrations, excessive stresses, or the presence of cracks [147,148,149,150,151,152,153,154,155]. Although earth-based mortars tend to exhibit localized damage, self-sensing earth–cement composites displayed favorable electrical, diagnostic, and sensing properties during failure tests on molded casts [151]. While 3D-printable self-sensing cementitious composites are feasible, it is important to note that fiber orientation and inter-layer bonding can significantly impact the piezoresistive performance [148].

Employing computer-aided arrangements for ceramics presents an effective way to automate the construction process. An example was the initiative undertaken by the ETH Zürich group, where a permeable façade designed to regulate the ingress of air and sunlight was created [21]. This approach was to enhance winery conditions, aligning with functional requirements. The use of computer-aided assembly for eco-friendly products streamlines construction and reduces manpower and material waste. Integrating these technologies can accelerate construction timelines while promoting sustainability through environmentally friendly materials and methods.

There is a gap in the potential of 3D-printed materials. The existing studies focus only on the concept without showing scientific data or proof of the efficiency of the solution. However, there is undeniable potential for 3D-printed materials for efficient solutions.

## 5. Conclusions

Research aimed at optimizing the additive manufacturing of earth-based mortars has been on the rise in the past decade. The growing interest in modern construction with raw clay has gained significant importance due to the sustainability of the industry. Clay is readily available worldwide and reduces construction waste, since it can be reused without any additional processing or easily recycled. Additionally, earth-based materials have the potential to enhance efficiency by regulating temperature and relative humidity in indoor spaces, as well as capturing pollutants, therefore contributing to improve IAQ and the comfort and health of the occupants. The development of 3DPEM with functional performance adds value to this new construction process. In this context, it is possible to refer to additive manufacturing with earth as 4DPM (fourth-dimension printable mortar). However, rapid construction is a key characteristic of this manufacturing approach, and the rate of building is controlled by the development of printed product strength. In the case of clay-based mortars without additions, the mechanical strength in the fresh state tends to be relatively low and only achieved after drying, which takes time. Studies in the literature have shown that the maximum wall height achievable is typically less than 1 m per day. To expedite the setting process, some research groups and companies have added hydraulic binders, but this approach significantly increases the ecological footprint of the printed mortars. Only two references have suggested the use of bio-based fast-setting alternatives. Therefore, there is a need for further research to improve the fresh strength of these mortars using ecologically friendly additives. While a limited number of studies of 3D-printed earth mortars have explored the mechanical and structural performance of elements on a large scale, a significant volume of publications has focused on small-scale tests.

Out of the 42 experimental studies reviewed, only 35% provided information about the type of clay, and details regarding the chemical or mineralogical composition were available in only 17% of the published studies. This lack of information concerning earth mortar composition hinders the ability to comprehensively analyze results and initiate a round robin testing approach. Moreover, complete information about the system and printing settings is often missing.

Due to the absence of standardized international testing procedures, printed earth mortar characterization remains non-uniform. Different tests are performed, and most publications primarily focus on printability without thoroughly assessing workability and mechanical performance. As of now, in terms of fresh performance, 75% of experimental studies exclusively concentrate on printing material. In most cases, the results of printability tests are not presented; characteristics such as pumpability, extrudability, and buildability are missing. Demonstrating that earth is a viable printable material has had a limited impact on the advancement of the technique. It is of greater importance to disseminate successful procedures and learn from unsuccessful attempts, but more characterization and established tests are needed.

For the success of the extrusion, the mortar needs to be compatible with the feeding system. As mentioned before, piston pump systems allow stiffer mortars due to the reduced friction and higher-pressure range, but this system is highly dependent on human interaction. At least 40% of published works reported the use of an exclusive progressive cavity, or Archimedes’ screw, which allows the comparison of yield stress, viscosity, and pumping pressure necessary to develop a printable mortar. For large-scale production, the conveying system should guarantee the constant flow and production of mortar.

Given these conditions and the available equipment on the market, a mixing pump or screw pump with a deposit seems to be a good solution for large-scale construction of building elements and houses. The literature is almost divided between two different delivery systems, each associated with a distinct plastic consistency. In terms of plastic mortars, more clay content is necessary, which can influence shrinkage negatively but positively increase buildability. Due to the limited number of experimental articles available, it is not possible to definitively determine which conveying system is more suitable for large-scale clay constructions.

It is intended that this review serve as a support for future research and provide critical and important information about what needs to be further studied in this field. Future research should frame the material properties and functionality of printed solutions. Understanding the technique and the relationship between material and system is crucial for the future and progress of low-embodied energy additive manufacturing.

## Figures and Tables

**Table 2:** The bibliometric output focuses on experimental approaches related to additive manufacturing utilizing raw earth/clay material, arranged in chronological order.

Reference	Explored Aspects *	Source **	Location	Group/ Company ***	Material ****	Type of Clay	Fibers	Additions *****
[36]	1	a	Morocco	WASP	LE	-	-	Unspecified type
[17]	1	c	Switzerland	ETH	LC	-	Cellulose, wood chips	Lime (unspecified type)
[37]	1	a	Spain	IAAC	LE	-	-	Bio additives
[27]	5	a	Italy	WASP	LE	-	Straw fibers	CL, HL
[38]	1, 3	d	UK	Cardiff	LE, P, BC	Kaolin/ball clay	Straw fibers	Unspecified type
[39]	1	b	Spain	IAAC	LE	-	-	Bio additives
[40]	1, 2	b	Cyprus	UCY	P	-	-	Unspecified type
[41]	1, 3, 4	b	Spain	IAAC	LE	-	Fibers (unspecified type)	Bio additives
[42]	1, 2, 3	a	Spain	IAAC	C	-	-	Bio additives
[28]	1, 2, 3, 5	a	Italy	WASP	LE	-	Straw fibers	CL, HL
[43]	1, 2	c	UK	P., A., C.	LE, P, BC	Kaolin/ball clay	Straw fibers	-
[44]	1, 2	b	France	Bretagne Sud	LE	Kaolinite, illite, smectite	-	SA
[45]	1, 3	c	France	LGCgE	E	Kaolinite, illite	Short fibers, flax shives	OCP
[46]	1, 5	c	Spain	IAAC	E	-	-	Bio additives
**Reference**	Explored Aspects *	Source **	**Location**	**Group/** Company ***	Material ****	**Type of Clay**	**Fibers**	Additions *****
[47]	1, 2	c	France	INSA Rennes	KB; RB; SB	Kaolin, illite, chorite, smectite	SB: flax fibers and woven flax fiber fabric	SHMP
[48]	1, 2	a	The Netherlands	4TU	E	-	Straw, jute, and hay	-
[49]	1, 2	a	Spain	IAAC/WASP	LE	-	Straw fibers	CL, HL
[32]	5	a	USA	EO	-	-	-	-
[50]	1	b	UK	P., A., C.	LE, P, BC	Kaolin/ball clay	Straw fibers	-
[51]	1, 3	b	UK	P., A., C.	LE	-	Straw fibers	-
[52]	1, 2	b	Cyprus	UCY	E	-	Straw fibers	-
[53]	1, 2	b	Cyprus	UCY	P; LC; LE	Kaolin	Straw fibers	NaCl, SHMP
[54]	5	a	USA	EO	-	-	-	-
[55]	1	b	UK	P., A., C.	LE	-	Straw fibers	-
[56]	2	b	UK	P., A., C.	LE	-	Straw fibers	-
[29]	1, 5	a	Italy	WASP	LE	-	Rice husk	-
[30]	5	a	Germany	WASP	LE	-	Rice husk and straw	-
[33]	5	d	USA	EO	-	-	-	-
[57]	1, 2	b	Italy	WASP	LE	Calcareous loam	Rice husk, polypropylene	CL, HL
[31]	5	d	Dubai	WASP	-	-	Fibers (unspecified type)	-
[18]	1, 2, 5	a	Switzerland	ETH	C	-	-	-
[58]	1, 2	b	Italy	WASP	LE	Calcareous loam	Rice husk, polypropylene	CL, HL
[59]	1, 2	b	USA	Park	C	-	Straw fibers	HL
[60]	1, 2	c	USA	Columbia	LE	Kaolinite and illite	Straw fibers	SA, MC
[61]	1, 2	b	Peru	PUCP	LE	Kaolinite and illite	Straw fibers	Potato starch
[62]	1, 3	b	France	LGCgE	E	Kaolinite, illite	-	OCP, CSAC, FA, CAM
[63]	1, 2	b	Israel	IIT	CC	Kaolinite	Hemp shiv; cellulose; horse manure	-
[26]	1, 2	b	France	IMT	LE	Kaolinite and illite	-	OCP, MP; SP; PCE
[64]	1, 2	b	France	L2MGC	LE	-	Flax fibers	-
[65]	1, 2, 4	b	Italy	UP	LE	-	Rice husk; jute, coconut; sisal fibers; goat hair	OCP; HL; municipal solid waste bottom ash

Notes: * Explored aspect: (1) Workability and design; (2) Mechanical/Structural properties; (3) Thermal performance; (4) Environmental performance/Life cycle assessment (LCA); (5) In situ building performance. ** Source type key: (a) Online article; (b) Journal article; (c) Conference paper; (d) Online media. *** Group/company: 4TU—4TU.Federation; Bretagne Sud—University Bretagne Sud; Cardiff—Cardiff University; Columbia—Columbia University; EO—Emerging Objects; ETH—Swiss Federal Institute of Technology Zurich; IAAC—Institute for Advanced Architecture of Catalonia; IIT—Israel Institute of Technology; IMT—Institut Mines-Télécom; INSA Rennes—Institut National des Sciences Appliquées de Rennes; L2MGC—The Mechanical Laboratory of Civil Engineering Materials; P., A., C.—University of Plymouth, University of Adelaide and Cardiff University; Park—Park University; PUCP—Pontificia Universidad Católica del Perú; UCY—University of Cyprus; UP—University of Padova; WASP—World’s Advanced Saving Project. **** Earth material: Earth, unknown location (E), Local earth, local source soil (LE); Porcelain (P); Ball clay (BC); Local brick factory clay mixture (LC); Clay-based soil Kaolins de Bretagne (KB); Local soil extracted in Redon Brittany (RB); Local soil extracted in Saint-Sulpice-La-Forêt Brittany (S); Clay (C). ***** Addition: Hydrated lime (CL); Hydraulic lime (HL); Ordinary Portland cement (OCP); Calcium sulfoaluminate cement (CSAC); Fly ashes (FA); Sodium alginate (SA); Microdrystaline cellulose (MC); Sodium chloride (NaCL); Sodium hexametaphosphate (SHMP); Citric acid monohydrate (CAM); Modified phosphonate (MP); superplasticizer polymer (SP); Polycarboxylate (PCE).

**Table 3:** Bibliometric output focuses on theoretical approaches or review papers related to additive manufacturing with raw earth/clay material, arranged in chronological order.

Reference	Explored Aspects *	Source **	Location	Group/Company ***
[66]	5	b	Portugal	CIAUD
[67]	4	b	UK	C., A., T.
[68]	5	b	USA	EO
[69]	1, 2	a	Spain	IAAC
[70]	3, 4	c	Germany	TUDelft
[71]	3, 4	c	USA	AFIT
[72]	1, 2, 3, 4	b	Germany	Collaborations
[73]	1, 2	b	Portugal	FEUP
[74]	1, 2	b	Germany	TU Darmstadt
[35]	1, 2, 3, 4	b	USA	RMIT, C., A.
[75]	1, 2	b	Switzerland	ETH
[76]	1, 2	b	USA	Penn State

Notes: * Tested aspect key: (1) Workability and design; (2) Mechanical/Structural properties; (3) Thermal performance; (4) Environmental performance/Life cycle assessment (LCA); (5) In situ building performance. ** Source type key: (a) Online article; (b) Journal article; (c) Conference paper; (d) Online media. *** Group/company: AFIT—Air Force Institute of Technology; CIAUD—Research Centre for Architecture, Urbanism and Design/University of Lisbon; C., A., T.—Cardiff University, University of Adelaide and Taibah University; Collaborations—German universities and TUDelft; EO—Emerging Objects; ETH—Swiss Federal Institute of Technology Zurich; FEUP—Faculty of Sciences of the University of Porto; IAAC—Institute for Advanced Architecture of Catalonia; Penn State—The Pennsylvania State University; RMIT, C., A.—RMIT University, Cardiff University and University of Adelaide; TU Darmstadt—Technical University of Darmstadt; TUDelft—Delft University of Technology.

**Table 4:** Laboratory tests characterizing the clayey earth and mortar, in the fresh and hardened state, and the pump system used, arranged in chronological order.

Reference	Year	XRD/XRF *	Rheology **	Fresh Properties	Hardened Properties	Pump System
[36]	2014	-	-	Printability	Test not described	Screw pump
[17]	2014	-	-	Buildability	Test not described	Remote deposition
[37]	2015	-	-	Printability	Test not described	Piston pump
[27]	2016	-	-	Printability	Test not described	Screw pump
[38]	2017	-	-	Printability	-	Piston pump
[39]	2017	-	-	Printability	Test not described	Piston pump
[40]	2018	-	-	Printability	-	Piston pump + screw pump
[41]	2018	-	-	Printability	3-point flexural test	Piston pump
[42]	2018	-	-	Printability	3-point flexural test	Piston pump
[28]	2018	-	-	Printability	-	Screw pump
[43]	2018	-	-	Printability	-	Piston pump
[44]	2018	XRD	RR	Yield stress, fresh strength, visual voids layer section, printability	Uniaxial compression test	Screw pump
[45]	2018	-	-	Penetration, Vicat needle, printability	Uniaxial compression test, shrinkage	Screw pump
[46]	2019	-	-	Printability	Test not described	Piston pump
[47]	2019	XRD	-	Extrusion	Uniaxial compression test, dry density	Screw pump
[48]	2019	-	-	Printability	-	Screw pump
[49]	2019	-	-	Printability	-	Screw pump
[32]	2019	-	-	Printability	-	Screw pump
[50]	2019	-	-	Printability	Tensile and compressive strength simulation, shrinkage	Piston pump
[51]	2019	-	-	Printability	Thermal conductivity tests	Piston pump
[52]	2020	-	-	Printability	-	Piston pump + screw pump
[53]	2020	-	-	Printability	-	Piston pump + screw pump Piston pump
[54]	2020	-	-	Printability	Test not described	Screw pump
[55]	2021	-	-	Pumpability, extrudability, buildability, inclined printing	Test not described	Piston pump
[56]	2021	-	-	Printability	Uniaxial compression test, shrinkage	Piston pump
[29]	2021	-	-	Printability	Test not described	Screw pump
[30]	2021	-	-	Printability	Test not described	Screw pump
[33]	2021	-	-	Printability	-	-
[57]	2021	-	RP	yes, not described	Uniaxial compression test	none
[31]	2021	-	-	Printability	Test not described	Screw pump
[18]	2021	-	-	Buildability	Uniaxial compressive strength, shrinkage	Smash
[58]	2022	-	-	Printability	Uniaxial compression test, shrinkage	Screw pump
[59]	2022	-	-	Plasticity test, printability	Uniaxial compression test, flexural strength test, splitting tensile test	Piston pump
**Reference**	**Year**	XRD/XRF *	Rheology **	**Fresh Properties**	**Hardened Properties**	**Pump System**
[60]	2022	XRD	-	Pumpability, extrudability, buildability	Uniaxial compression test, three-point flexural test	Piston pump
[61]	2022	XRF	SVT	Extrusion, buildability, Vicat needle, fresh strength	Uniaxial compression test, shrinkage, capillarity absorption test	Screw pump
[62]	2022	XRF	-	Penetrometer, Vicat needle, printability	Uniaxial compression test, shrinkage	Screw pump
[63]	2023	-	-	Material flow, extrudability, buildability	-	Piston pump
[26]	2023	XRF	-	Mini slump, mini fresh strength, flow table, Vicat needle, extrudability, printability	Uniaxial compression test, mercury intrusion porosity	Piston pump + screw pump
[64]	2023	-	-	Mini slump test, printability	Uniaxial compression test, three-point flexural test, apparent shear test	Not mentioned
[65]	2023	XRD	-	Printability	Uniaxial compression test, three-point flexural test, shrinkage	Piston pump + screw pump

* X-ray diffraction analysis (XRD); X-ray fluorescence (XRF). ** Rheology: Rotational Rheometer (RR); Rotational plate (RP); Shear vane test (SVT).

## Data Availability

Not applicable.
